# Supplementary material for: Neuraminidase Antibody Response to Homologous and Drifted Influenza A Viruses After Immunization with Seasonal Influenza Vaccines
Source: Vaccines (Basel). 2024 Nov 27;12(12):1334. doi: 10.3390/vaccines12121334 (PMC11680112; doi:10.3390/vaccines12121334)
Supplement: Supplementary file 1 [file vaccines-12-01334-s001.zip › vaccines-3300804-supplementary.pdf]

Table S1. Seroprotection levels to HA and NA of influenza viruses A/H1N1pdm09 and A/H3N2 before and after vaccination with seasonal IIVs in patients of different age groups (%).

Assay	Age	A/H1N1pdm09				A/H3N2			
		S1	S2	S1	S2	S1	S2	S1	S2
		Michigan/15		Gd-Maonan/19		Singapore/16		Brisbane/18	
HI	≥60	50.0	80.0	20.0	33.3	46.7	80.0	3.3	23.3
	<60	73.5	82.4	11.8	23.5	44.1	82.4	2.9	11.8
		H6N1/13		H6N1/19		H6N2/14		H6N2/18	
NI	≥60	40.0	46.7	6.7	23.3*	33.3	63.3	3.3	3.3
	<60	38.2	52.9	0.0	5.9*	41.2	61.8	0.0	2.9

* - P<0.05, Fisher's exact test.
